# A time motion study of manual versus artificial intelligence methods for wound assessment

**DOI:** 10.1371/journal.pone.0271742

**Published:** 2022-07-28

**Authors:** Heba Tallah Mohammed, Robert L. Bartlett, Deborah Babb, Robert D. J. Fraser, David Mannion

**Affiliations:** 1 Swift Medical Inc., Toronto, ON, Canada; 2 Valley Wound Healing Centre Inc, Modesto, California, United States of America; 3 Arthur Labatt Family School of Nursing, Western University, London, ON, Canada; Indiana University Purdue University at Indianapolis, UNITED STATES

## Abstract

**Objectives:**

This time-motion study explored the amount of time clinicians spent on wound assessments in a real-world environment using wound assessment digital application utilizing Artificial Intelligence (AI) vs. manual methods. The study also aimed at comparing the proportion of captured quality wound images on the first attempt by the assessment method.

**Methods:**

Clinicians practicing at Valley Wound Center who agreed to join the study were asked to record the time needed to complete wound assessment activities for patients with active wounds referred for a routine evaluation on the follow-up days at the clinic. Assessment activities included: labelling wounds, capturing images, measuring wounds, calculating surface areas, and transferring data into the patient’s record.

**Results:**

A total of 91 patients with 115 wounds were assessed. The average time to capture and access wound image with the AI digital tool was significantly faster than a standard digital camera with an average of 62 seconds (P<0.001). The digital application was significantly faster by 77% at accurately measuring and calculating the wound surface area with an average of 45.05 seconds (P<0.001). Overall, the average time to complete a wound assessment using Swift was significantly faster by 79%. Using the AI application, the staff completed all steps in about half of the time (54%) normally spent on manual wound evaluation activities. Moreover, acquiring acceptable wound image was significantly more likely to be achieved the first time using the digital tool than the manual methods (92.2% vs. 75.7%, P<0.004).

**Conclusions:**

Using the digital assessment tool saved significant time for clinicians in assessing wounds. It also successfully captured quality wound images at the first attempt.

## Introduction

Chronic wounds are a highly prevalent condition that can significantly diminish patients’ quality of life and impose a financial burden on patients and the health care system [1]. The ubiquity and impact of chronic wounds has led to them being called the “silent epidemic,” affecting a large population in North America, and globally [1]. Evidence shows that careful assessment and continuous measurements of a wound using valid and reliable quantitative measurement methods are fundamental to wound care management [2–4]. Various factors such as discoloration, swelling, shape irregularity, and location can impact the ability to define and accurately measure wounds’ margins and depth [5, 6]. Wound assessment largely depends on evaluating the visual elements of the wound, such as the amount of surrounding erythema, the presence and color of granulation tissue, wound drainage, and accurately measuring the key dimensions of the wound to calculate the wound area and volume. These processes are typically accomplished by traditional methods, such as a photographic image of the wound using a digital camera, a paper-ruler, and a depth probe to determine the key measurements of the wounds [7]. However, using these traditional methods in daily practices is not ideal [8], especially when measuring irregular wound boundaries [9, 10], or unequal distances from the edge of the wound [11]. A growing evidence base considers these methods invasive, inaccurate, and time-consuming [12, 13].

Patient outcomes are positively associated with the amount of clinicians’ time dedicated to each patient’s care [14–17]. However, with the increase in clinicians’ documentation workload and emergent workforce shortages [17, 18], the amount of time available for different wound assessment activities is limited. Optimizing frontline efficiencies is essential to promoting practice capacity and high-quality wound care [19].

New wound assessment technologies with an advanced camera calibration could provide better image quality and additional information while saving time and money [13]. Moreover, several studies have highlighted that digital wound measurement methods are more reliable and improve wound assessment and documentation processes [10, 20]. For example, one study conducted in Toronto found the Swift Skin and Wound application (Swift) is more reliable in measuring wound area than the traditional ruler method (ICC = 0.97–1.00 vs. 0.92–0.97) [10]. Furthermore, the authors highlighted the challenges of using the ruler method in measuring islands of healing in the wound bed itself or its edges [10].

Swift is a non-invasive digital tool utilizing artificial intelligence (AI) to provide standardized wound assessments. The Swift app uses an FDA-registered fiducial marker to capture scientifically color calibrated images and uses intelligent features to automatically identify wound boundaries, record measurements, accurately calculate surface area, depth and immediately upload and document all this information in patients’ charts. Swift’s proprietary fiducial marker (HealX) provides true color imaging by compensating for ambient lighting bias. HealX is a small blue adhesive fiducial sticker placed beside the wounds as a point of reference upon evaluation of a wound.

**Fig 1:**
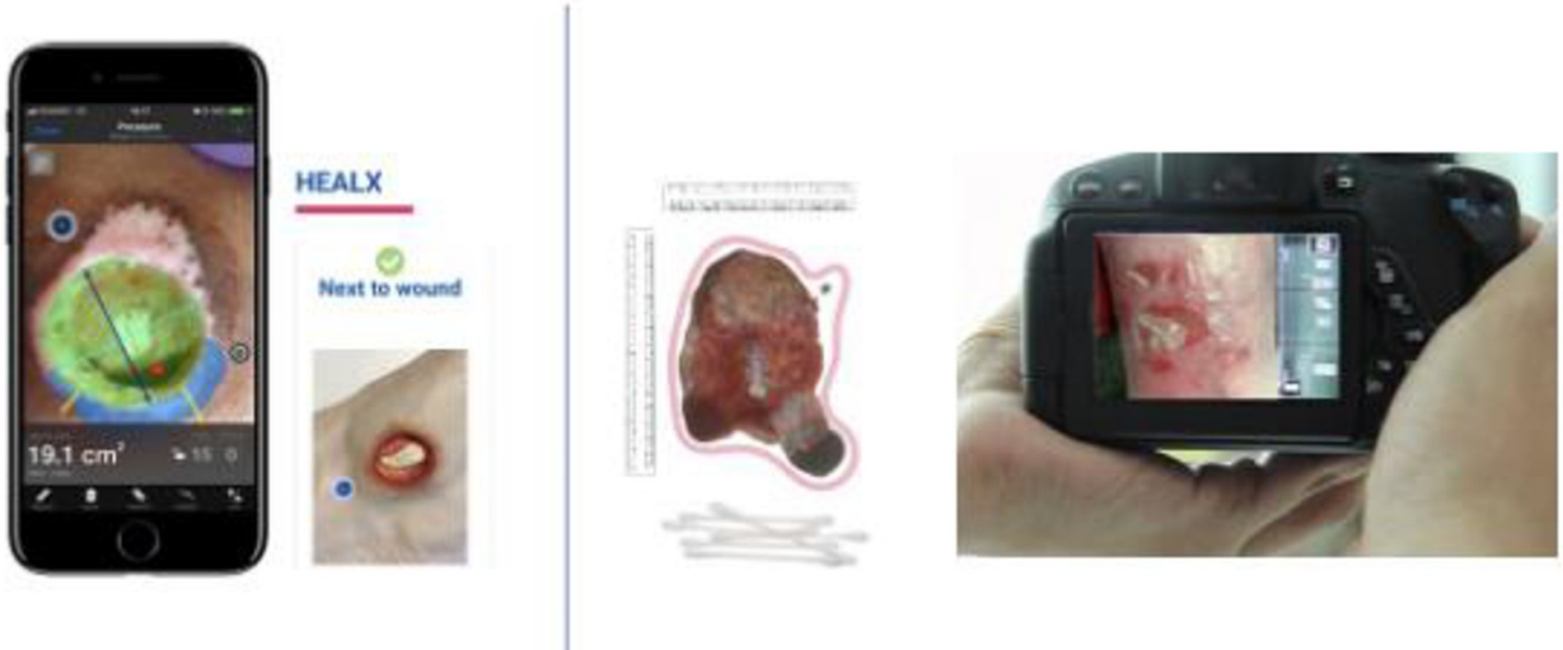
Swift software with fiducial marker (on the left) vs. traditional methods (right).

The precision-manufactured HealX design elements help the software calibrate the wound photo for color, lighting and size–all in real time on a smart device. Next, the software automatically and accurately traces the wound and calculates clinically validated measurements. HealX is foundational to ensuring the high accuracy and consistency of these wound image and wound measurements. It helps clinicians capture accurate consistent wound images regardless of the different lighting, body position, or camera setting (Fig 1).

Assessing wound areas in different illumination conditions and angles may exhibit color bias leading to incorrect assessments [21, 22]. The bias can range from too red (tungsten) suggesting infection, to blue hues (LED) suggesting ischemia, to green hues (fluorescent lighting) suggesting pseudomonas infection [23]. The FDA registered Swift fiducial marker compensates for bias and provides a registered distance perspective for comparable image sizing.

The app is designed to capture accurate surface area measurements even for irregular wounds, which cannot be accomplished using the traditional methods. Traditional length and width measurements assume all wounds have rectilinear geometry—which is not the reality.

Swift application provides equitable access to an AI standardized technology that can be used to assess wounds in large and small clinics in both urban and rural settings. Once downloaded, the Swift application does not require the internet to operate. The platform is interoperable with many healthcare systems and can be easily customized and adapt to the clinics’ workflow.

Despite the positive perception of integrating with a digital evaluation, published literature on the time saved is limited. Au and colleagues found in their pilot study that Swift technology is faster for measuring than manual methods [24]. For clinicians to actively adopt digital technology, these tools must fit within their existing workflows and provide time savings for measuring and charting wounds.

This research project was built on the pilot project conducted by Au and colleagues [24]. The study’s primary objective was to investigate the amount of time healthcare providers spent on wound assessments in a real-world environment. Specific activities required for wound assessments include labeling wounds, capturing images, measuring wounds, calculating surface areas, and transferring data into the patient’s record. A comparison was made using Swift Skin and Wound application vs. manual methods. The study also aimed at comparing the proportion of captured quality wound images on the first attempt by the assessment method.

## Material and methods

### Study design and setting

The quality improvement time and motion study was conducted at Valley Wound Healing Center (VWHC) in Modesto, California, US to study staff time and resource utilization. VWHC collected data on the time required for nurses to complete a wound assessment on chronic wound patients using manual methods vs. Swift. VWHC has operated since 1990 with over 50 years combined wound experience among their medical staff. VWHC is a self-standing outpatient wound clinic that accepts all types of wounds, including diabetic, venous stasis, arterial, pressure ulcers, surgical wounds, trauma wounds, skin tears, blisters, burns, etc. The centre receives an average of 130 wound patients per day, referred from other specialists, primary care providers, hospitals, and on occasion, self-referrals.

### Study participants and data sources

VWHC has seven medical assistants (MAs) and three licensed vocational nurses (LVNs). From a list of all nurses practicing at the VWHC, clinicians meeting the eligibility criteria were invited to join the study. The eligibility criteria allowed licensed clinicians of any age, gender and years of practice who can provide direct wound care and evaluation to patients using both methods during the weekly follow-up evaluation days at the center to participate in the study. Participation was voluntary.

Four MAs and one LVN met the inclusion criteria and agreed to join the study. They were asked to record the time needed to complete a wound evaluation for patients referred for routine wound evaluation on the weekly follow-up days at the clinic. Clinicians implied consent to participate in the study was established when they recorded their time on the data collection sheet provided to them and submitted to researchers. Clinicians were asked to include assessment time of wounds for patients 18 years of age or older with chronic active wounds, including diabetic, venous stasis, arterial, pressure ulcers, surgical wounds, skin tears, abscesses, blisters, and burns. A total of 115 wounds referred to the center on the follow-up days during the study period met the inclusion criteria and were assessed using both manual and digital methods. Wound assessments were carried out over a period of two weeks starting Jan 24, 2022.

No wound measurement data was collected. Patients were informed that the clinicians would document the time they spent assessing their wounds and inform researchers of the time spent, wound type, patient age, and gender. Clinicians only proceeded to document their time if the patient consented verbally and was comfortable with sharing the unidentifiable information.

To maintain confidentiality, each wound assessed in this study was assigned a study ID number by the nurses at VWHC. The study-medical record number (MRN) key was not shared at any point with the research team, leaving no possibility for the researchers to identify the patients. Assessment of wounds was part of the routine care provided at the weekly follow-up days at the clinic. Tracking time did not involve any particular intervention or preparation and did not impact the standard care provided to patients. The institutional board director approved the study protocol.

### Data collection process

Clinicians participating in the study were supplied with two data collection Excel ^TM^ spreadsheets that included all-wound assessment-related activities necessary to evaluate a wound using the manual methods and the Swift digital application downloaded on tablet or smartphone. The MAs used the spreadsheet to record time for each of the listed activities. In general, the clinicians used the stopwatch application on their phones to time each activity and then they recorded the time in the spreadsheet.

For the manual method, an administrator first prepared the labels necessary to register and assess patients manually. The labels contained the patient’s initials, wound number, and date of image. The labels were placed in the camera’s field of view. The LVN then recorded the time to complete the labelling process for each patient on the data collection spreadsheet.

For the visual assessment, the MAs used a traditional digital camera to capture images. The recorded time to capture an acceptable image started when the camera was touched and stopped when the image was captured, and the camera was placed back on the counter. The MAs also recorded how many pictures were taken before a clear, acceptable image was captured.

The LVN also recorded the time to transfer the camera SIM card to the computer, the time to review SIM card files, and the time to transfer files to the EMR. The time it took the MAs to measure the wound length and width using the manual paper-ruler method and the time to transfer the measurements to the system and to calculate wound surface area was also timed and recorded by the LVN.

**Fig 2:**
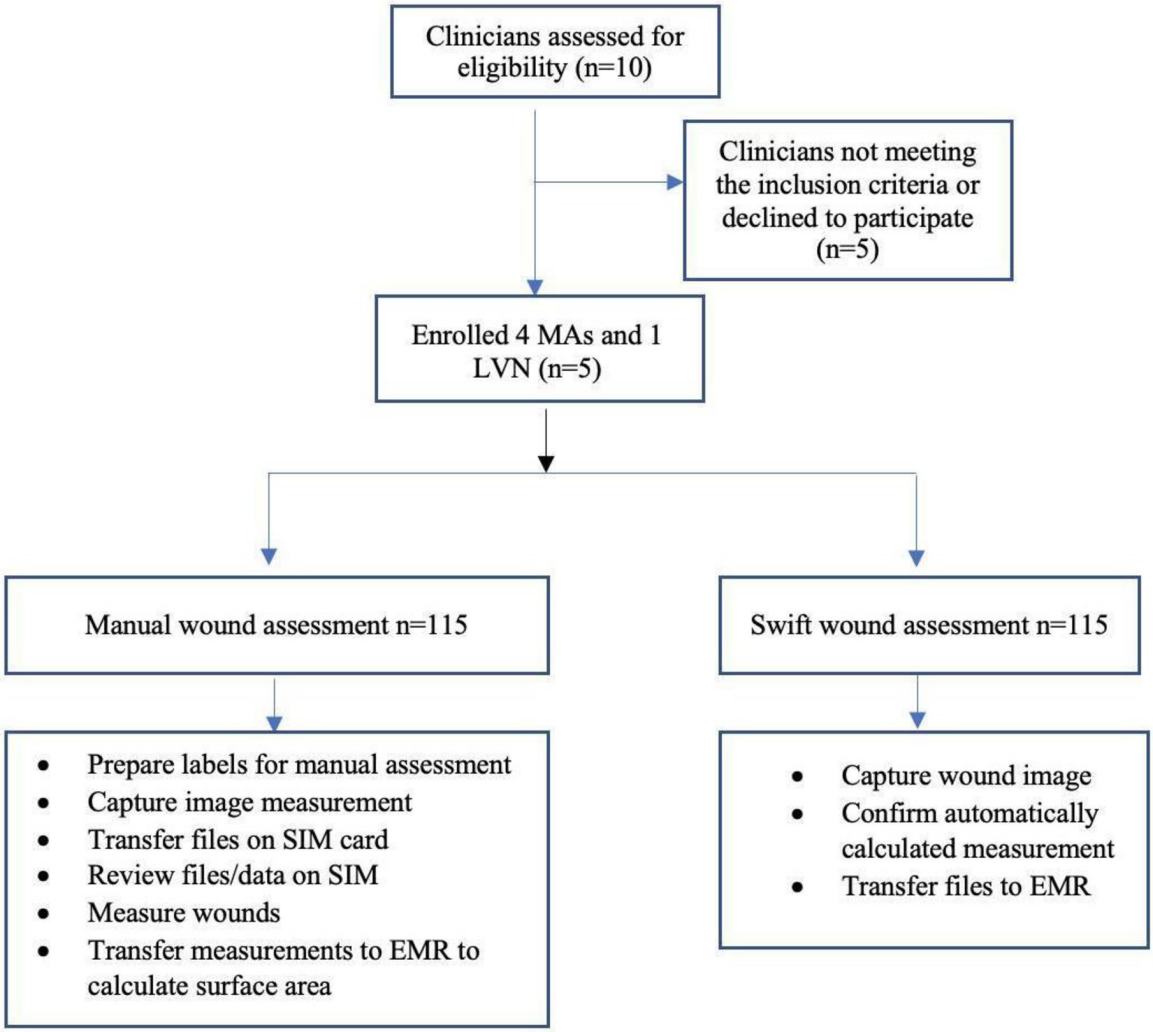
Enrollment and wound assessment workflow.

Using Swift for the wound visual assessment, the MAs used the built-in digital camera guided by the HealX adhesive sticker placed beside the wound to capture images. The recorded time started when the iPad was touched and the HealX sticker was placed. Recording the time stopped when an image was captured, and the iPad was placed back on the counter. The MAs also recorded how many pictures were taken before a clear, acceptable image was captured using Swift. For the measurements, the staff recorded the time it took to confirm the measurements and wound surface area automatically calculated by the application. Then the time to transfer the information from the Swift application to VWHC’s EMR was recorded (Fig 2).

To ensure consistency, a sample of eligible ten patients with 14 chronic wounds was selected at random, and each nurse was asked to evaluate the same 14 wounds independently and record the time for each activity needed for the wound assessment using both manual and digital methods. The level of inter-rater agreement on the time required to assess patients by methods was measured. The nurse’s total time was compared to ensure uniformity.

### Statistical analysis

Data was analyzed using the Statistical Package for Social Sciences (SPSS) (SPSS, IBM Corp, Armonk, NY. Version 28; 2022). The descriptive analysis generated from the numeric age variable and categorical gender, and type of wounds variables were calculated and displayed as frequencies, mean and standard deviations.

An intraclass correlation coefficient (ICC) test was used to determine if there was an agreement between the clinicians’ recorded time of different wound assessment activities using manual and digital methods for the 14 wounds. ICC was interpreted based on the Koo and Li, 2016 guidelines where measurements below 0.50 are poor, between 0.50 and 0.75 are considered moderate, between 0.75 and 0.90 are good, and above 0.90 are excellent [25].

Additionally, bivariate analyses were conducted, and Paired samples t-test was used to examine whether there was a mean difference in the time to evaluate wounds using a traditional manual method versus Swift’s digital application.

The Chi-square and Fisher Exact tests were used to examine whether there was a statistically significant difference in pictures taken once or more to get a clear, acceptable image in the relation to the method of evaluation (manual vs. Swift) per the type of wound. A two-way repeated measure ANOVA was computed to determine the effect of the type of wound on the time required to evaluate the wound using different assessment methods. A 2-sided P-value of <0.05 was considered statistically significant.

## Results

### Overall characteristics of clinicians participated in the study and inter-rater reliability

Recording the time needed to assess patients’ wounds was conducted by four MAs and one LVN. All clinicians were females and their years of practice ranged from 3 to 10 years, with a mean of 5.9 years.

ICC demonstrated a good agreement between clinicians in their recorded time of manually capturing images, ICC = 0.868, (95% CI, 0.76–0.96), P<0.001 and manually measuring wounds ICC = 0.834, (95% CI, 0.78–0.94), P<0.001.

Moreover, ICC demonstrated a moderate agreement between clinicians in their recorded time of capturing images using Swift, ICC = 0.552, (95% CI, 0.52–0.73), P<0.001 and measuring wounds using Swift, ICC = 0.581, (95% CI, 0.53–0.75), P<0.001.

### Overall characteristics of patients included for wound assessment at VWHC

A total of 91 patients were assessed for the study. 49 (53.8%) were females and 42 (46.2%) were males. The age of patients ranged from 18 to 90 years, with a mean of 62.8 years.

**Fig 3:**
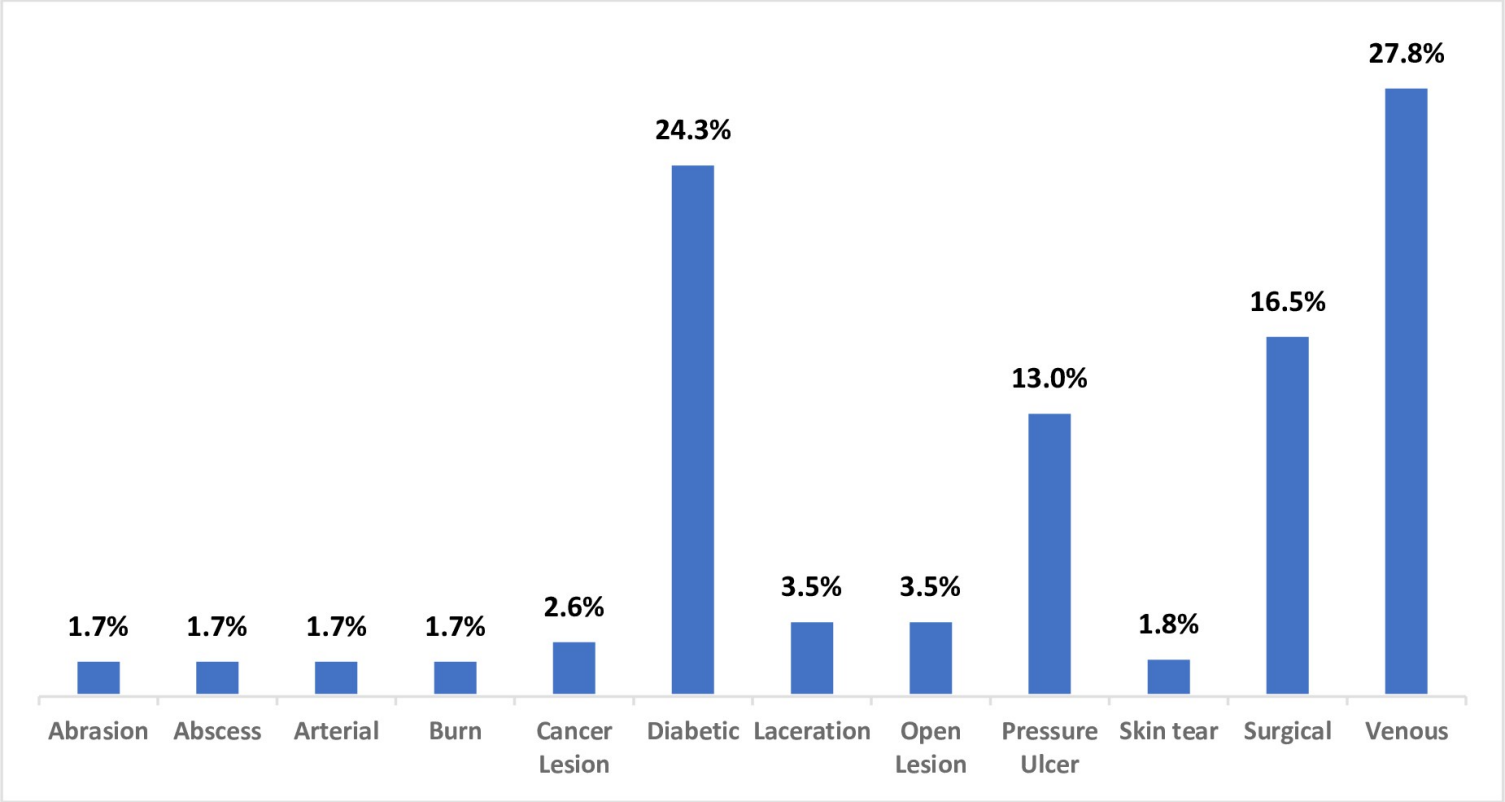
Types of wounds assessed using manual and Swift methods.

A total of 115 wounds were identified with a wide range of wound types. Of this total, 32 (27.8%) were venous, 28 (24.3%) were diabetic, 19 (16.5%) were surgical (Fig 3).

### Number of attempts needed to acquire a clear wound image using manual and Swift methods

Overall, acquiring a clear acceptable wound image was significantly more likely to be achieved the first-time using Swift compared to the manual methods (92.2% vs. 75.7%, P<0.004).

The proportion of captured wound images on the first attempt is consistently higher with Swift compared to the manual methods for the different types of wounds. All pressure ulcers (100.0%) were captured with the first picture using Swift compared to only 73.3% using manual methods. However, no significant differences were detected in relation to the evaluation method for the different types of wounds (Table 1).

**Table 1:** Number of times pictures was taken until deemed acceptable by clinicians.

	Manual methods	Swift	P value
	N = 145	N = 115	
	N (%)	N (%)	
**Number of pictures taken until acceptable**			
Once	87 (75.7%)	106 (92.2%)	P<0.004*
More than one time	28 (24.3%)	9 (7.8%)	
**By wound type**			
**Diabetic ulcer**	N = 28	N = 28	
Once	20 (71.4%)	25 (89.3%)	P = 0.177
More than one time	8(28.6%)	3 (10.7%)	
**Pressure ulcer**	N = 15	N = 15	
Once	11 (73.3%)	15 (100.0%)	P = 0.100
More than one time	4(26.7%)	0 (0.0%)	
**Surgical wound**	N = 19	N = 19	
Once	16 (84.2%)	18 (94.7%)	P = 0.604
More than one time	3(15.8.%)	1(5.3%)	
**Venous ulcer**	N = 32	N = 32	
Once	23 (71.9%)	29 (90.6%)	P = 0.107
More than one time	9(28.1%)	3(9.4%)	

### Clinician time to complete different activities necessary to evaluate wounds using manual and Swift methods

The average time needed to complete each activity necessary to successfully assess a wound is presented in Table 2. Overall, the average time to capture and access the wound image with Swift was significantly faster by 78% with an average of 62 seconds (P<0.001).

**Table 2:** Time to successfully complete each activity necessary to evaluate wounds.

	Manual (N = 115) Mean (mm.ss.ss)± SD	Swift Mean (mm.ss.ss)± ± SD	
**Capture wound image**			
**Average time to capture and transfer pictures to system**	1.31.15 ± 00.20	00.29.38± 00.16	**P<0.001**
**Wound Measurements**			
** Average time to measure wound length and width**	00.26.28± 00.22	00.25.05± 00.16	P = 0.434
**Total time to calculate surface area**	00.44.22± 00.00	00.25.05± 00.16	**P<0.001**
**Total time to measure and calculate surface area**	01.10.10± 00.22	00.25.05± 00.16	**P<0.001**
**Total workflow**			
**Total workflow**			
Label/ image /transfer image /measure/ calculate wounds	02.53.15± 00.38	00.54.44± 00.26	**P<0.001**
**Total workflow**			
Label/ image/ transfer image /measure/ calculate/upload measurements	02.53.15± 00.38	01.52.19± 00.26	**P<0.001**

The average time to measure a wound’s length and width was similar for the manual and Swift methods, with both taking an average of 25–26 seconds. However, with Swift calculating the surface area automatically, the digital method was significantly faster by 77% at accurately measuring and calculating the wound surface area with an average of 45.05 seconds (P<0.001).

Overall, the average time to complete a wound assessment using Swift was significantly faster by 79% with an average of 2.39 minutes than manual methods (0.54.44 vs. 2.53.15, P<0.001).

In total, slightly more than half of the clinicians’ time (54%) was saved assessing 115 wounds using Swift with 2:44:20 hours spent on wound evaluation vs. 5:31:21 hours spent using manual methods.

### Time to complete different activities necessary to evaluate wounds using manual and Swift methods by wound type

Table 3 summarizes the average time needed to complete each activity to effectively complete a wound assessment by the type of wound. Overall, the average time to capture and access images with Swift was significantly faster for all types of wounds compared to the manual method (P<0.001). The reduction in average time to complete a visual assessment using Swift was the highest for pressure ulcers with an average saving of 1.10.04 minutes. The time to assess surgical wounds was 1.03.32, venous ulcers was 1.03.12 and diabetic ulcers was 1.00.11 minutes.

**Table 3:** Time to successfully complete each activity necessary to evaluate wounds using manual and Swift methods by wound type.

	Average time to capture image	Average time to measure and calculate wounds	Average time to complete an assessment	Average time to compete and assessment and upload documentation
	Mean± SD	Mean± SD	Mean± SD	Mean± SD
**Diabetic Ulcer (N = 28)**				
Manual	01.30.57 ± 00.12	01.12.29± 00.20	02.54.35± 00.30	02.54.35± 00.30
Swift	00.30.46± 00.13	00.25.70± 00.14	00.56.16± 00.21	01.54.31± 00.22
	**P<0.001**	**P<0.001**	**P<0.001**	**P<0.001**
**Pressure Ulcer (N = 15)**				
Manual	1.36.01 ± 00.38	01.08.47± 00.21	02.58.38± 00.55	02.58.38± 00.55
Swift	00.26.37± 00.15	00.26.50± 00.13	00.53.26± 00.23	01.51.01± 00.23
	**P<0.001**	**P<0.001**	**P<0.001**	**P<0.001**
**Surgical Ulcer (N = 19)**				
Manual	1.28.34 ± 00.09	01.07.16± 00.15	02.47.38± 00.22	02.47.38± 00.22
Swift	00.25.42± 00.15	00.24.37± 00.20	00.50.38± 00.34	01.47.56± 00.21
	**P<0.001**	**P<0.001**	**P<0.001**	**P<0.001**
**Venous Ulcer (N = 32)**				
Manual	1.32.23 ± 00.09	01.11.13± 00.29	02.55.22± 00.51	02.55.22± 00.51
Swift	00.29.11± 00.18	00.23.15± 00.21	00.51.36± 00.24	01.49.11± 00.24
	**P<0.001**	**P<0.001**	**P<0.001**	**P<0.001**

The average time to capture wound measurements and calculate wound surface area was significantly faster with Swift for all the wound types (P<0.001).

Overall, the average time to complete a wound assessment using Swift was significantly faster than using manual methods, resulting in an average savings of 2 minutes of clinicians’ time per wound across all wound types.

A two-ways repeated measures ANOVA test was computed to determine the effect of wound etiology on the time required to assess wounds using different assessment methods. No statistically significant differences were detected for the two-way interaction between assessment methods for different types of wounds, P = 0.061. However, testing the main effect of assessment methods only showed a statistically significant difference in the time spent assessing wounds between the manual vs. the Swift methods, F = 82.46, P<0.001. Therefore, the time it took clinicians to assess wounds was most likely impacted by the assessment method only even if the type of wounds being assessed changed.

## Discussion

The study’s primary aim was to quantify the time clinicians saved completing wound assessments using the Swift application vs. manual methods in a real-world clinical setting. The study also compared the need to repeat the capture of wound images in order to get a clear, acceptable picture of the wound by the assessment method. Our findings showed that the AI- based assessment tool was twice as fast. Using Swift automation, the staff completed all steps in about half of the time (54%) normally spent on wound manual evaluation activities. These activities include capturing wound images, transferring to EMR, measuring and calculating wounds.

The observed time savings in wound assessment activities can be linked to potential cost savings. Our study found an average time saving of 1.01–2.39 minutes per wound assessment using Swift compared to the traditional manual methods. So, for a total of 130 wound assessments per day, there is a potential average saving ranging between 21.8–51.7 days of clinicians’ time a year which is one to two months based on a 5 workdays/week. Swift digital tool does not directly profit from reducing clinicians’ time and organizations’ return on revenue. Using digital wound assessment tools in healthcare settings is the reasonable path to adequately use clinicians’ time and leverage their skills toward enhancing patient care. Reducing the time wasted on mechanical tasks and using standardized practice tools would translate into more focused consults with time better spent on wound management and improving care plans rather than performing mechanical tasks that can easily use up the consult time. Therefore, using Swift would improve clinical throughput and provide extra time for clinicians to care for a greater number of patients. This would potentially improve access to specialty care and reduce patient wait times. Our observations aligned with other studies that explored improvement of clinicians’ efficiency and capacity. Previous studies stated that practices that make clinicians more efficient and save them time are highly valued by healthcare organizations [26]. Even modest efforts that led to savings of mere minutes a day translated into improved clinician capacity and quality of care in the long term [26]. Jeffrey Farber and colleagues reported that an average of 7 minutes added to each 30-minute visit can add an extra 7–10 hours to a clinician’s workload per week [26–28]. This increased workload and decreased capacity can result in fewer patients and lost visit revenue [26].

When designing a digital health tool in healthcare, the technology must meet the user’s needs for better utilization and performance impact. Human factors such as visual perception, color discrimination, luminous and contrast discrimination, motor grips, etc., are important contextual factors to efficient device interface and usability characteristics [29]. According to the American National Standards Institute and Association for the Advancement of Medical Instrumentation, human factors are the elements that impact physical, sensory, emotional and intellectual capabilities [30]. Growing evidence substantiates the importance of ease of use, professional satisfaction, and technology interoperability in optimizing efficiency [31]. Swift surveyed clinicians across the US and Canada and assessed their level of satisfaction with Swift workflows and features. Of the 245 responses, 81% were satisfied with the technology, 79% would recommend it to others, and 77% believed Swift meets their clinical needs (unpublished data).

Furthermore, Swift provides an automated approach that improves time to assess wounds while facilitating quality photographic evidence of the wounds healing progress. Our findings showed that clinicians successfully captured quality images of the wounds on the first attempt, 16.5% more often with Swift than manual methods. Evidence shows that quality images are essential to accurately measuring the wound area and tracking wound progress [7], an attribute that can support successful pre-authorization claims [32].

Unlike Swift’s application, using standard digital cameras to capture wound images can present challenges in capturing quality images due to a lack of image calibration that accounts for variable lighting conditions, skin tones and distance the picture is taken from the wound. Altered colours and suboptimal images data common with the standard digital cameras reduces their reliability in supporting effective wound evaluation [21, 33–35].

Pre-authorization of coverage claims, especially for chronic non-healing wounds, can be challenging and differences in processing focus on the clinical criteria of the rendered wound service [32]. However, clinicians at VWHC stated that Swift’s quality wound images supported claims for insurance payments, with fewer or no denials for reauthorization requests. Langemo and colleagues attested that wound documentation is critical to tracking patients’ progress and demonstrating medical necessity [36]. They stated that wound images are complementary to written reports. Therefore, quality photographic documentation of wounds assists healthcare facilities in accurately measuring wounds, determining wound stage, assessing progress, selecting treatment, verifying the facility’s adherence to assessment best practices and supporting reimbursement processes [36].

Manual documentation is an inefficient process, consumes a large portion of clinicians’ time and can lead to provider burnout [37, 38]. Evidence shows that nurses spend between 25–41% of their time on paper and electronic documentation and review [37, 39, 40].

Reliable wound analytics and documented data are essential to wound assessment practice [19]. They provide clinicians with a holistic view of a patient’s health status and determine a wound healing trajectory required to develop a quality treatment plan [19]. Accurate assessment of the wound healing trajectory is important in determining efficacy of advanced therapies [41]. AI-powered technology uses a more standardized format, contents and plan, making it more reliable, accurate, and objective [42].

While Swift has established integrations with many Electronic Health Records (EHR) systems, the VWHC EHR and the Swift app were not integrated at the time of the study. Without integration, clinicians had to manually search for their patients in Swift before beginning an assessment and then manually type their notes into the EHR at the end of the assessment. Even without an integration between the Swift app and VWHC’s EHR, our finding still demonstrated a 40% faster workflow with an average of 1.38 minutes savings for the total evaluation process with Swift.

Enhancing the documentation process is complex and requires a robust solution to achieve superior outcomes [19]. Interoperability of technology can reinforce the benefits of quality technology and maximize its efficiency. Swift is designed as an Application Program Interface (API) which allows for integration into electronic medical records in the hospital, home health and community setting enabling automatic registration of patients and direct data sharing (e.g. PDF reports, discrete data) to be embedded into the patient records.

### Limitations

This study provides an insight into the time savings clinicians’ have experienced evaluating wounds using Swift-digital AI-powered wound assessment tool compared to the traditional manual methods. The study included 115 different types of wounds and assessed the impact on the same patients using both methods. However, the data set is still limited to wounds evaluated at one ambulatory outpatient clinical setting in the US, limiting the generalizability of our findings. It is worth noting that the number of wound referrals per day to this establishment is large, and the workflow is comparable to other wound care organizations. However, our population could differ from a more complex and mobility-restricted inpatient population.

Our conclusions are drawn from recording the time clinicians spent assessing wounds by the method. The study recruited clinicians who have been assessing wounds for 3–10 years and are quite professional in using the digital methods at their practice. The level of experience and knowledge of using the digital tools might differ at each practice, impacting the estimated time to assess wounds.

Our study used a within-subject design where the same wounds were evaluated using manual and Swift digital tools. Four independent clinicians conducted this approach. However, due to the clinic workflow, the same wounds were sometimes assessed by different clinicians rather than the same one. However, as the concordance on time spent on evaluating patients had little variability among the clinicians, we do not anticipate it affected our recorded time.

## Conclusions

Many clinicians’ have moved towards using digital tools within their practice settings to improve workflow and capacity. Using Swift saved significant time for clinicians in assessing wounds. It also facilitated a successful capture of quality images of the wounds at the first attempt. Our findings showed modest savings in clinicians’ time with Swift when transferring and charting wound files were considered. This highlights the necessity of following a model where digital tools are fully integrated within the healthcare system for more observed savings in clinicians’ time and improved capacity. As more clinicians use Swift as the assessment tool to evaluate wounds, more research focusing on time saved to measure and calculate the depth and volume of wounds would provide a broader vision of the capabilities of the tool’s enhanced features in evaluating wounds.

## Supporting information

S1 Data Time to wound assessment. (XLSX)
